# Patient Satisfaction with Hospital Inpatient Care: Effects of Trust, Medical Insurance and Perceived Quality of Care

**DOI:** 10.1371/journal.pone.0164366

**Published:** 2016-10-18

**Authors:** Linghan Shan, Ye Li, Ding Ding, Qunhong Wu, Chaojie Liu, Mingli Jiao, Yanhua Hao, Yuzhen Han, Lijun Gao, Jiejing Hao, Lan Wang, Weilan Xu, Jiaojiao Ren

**Affiliations:** 1 Department of Social Medicine, School of Public Health, Harbin Medical University, Harbin, Heilongjiang Province, China; 2 Department of Health Policy and Hospital Management, School of Public Health, Harbin Medical University, Harbin, Heilongjiang Province, China; 3 School of Psychology and Public Health, La Trobe University, Melbourne, Australia; 4 The Forth Affiliated Hospital of Harbin Medical University; Old Dominion University, UNITED STATES

## Abstract

**Objective:**

Deteriorations in the patient-provider relationship in China have attracted increasing attention in the international community. This study aims to explore the role of trust in patient satisfaction with hospital inpatient care, and how patient-provider trust is shaped from the perspectives of both patients and providers.

**Methods:**

We adopted a mixed methods approach comprising a multivariate logistic regression model using secondary data (1200 people with inpatient experiences over the past year) from the fifth National Health Service Survey (NHSS, 2013) in Heilongjiang Province to determine the associations between patient satisfaction and trust, financial burden and perceived quality of care, followed by in-depth interviews with 62 conveniently selected key informants (27 from health and 35 from non-health sectors). A thematic analysis established a conceptual framework to explain deteriorating patient-provider relationships.

**Findings:**

About 24% of respondents reported being dissatisfied with hospital inpatient care. The logistic regression model indicated that patient satisfaction was positively associated with higher level of trust (OR = 14.995), lower levels of hospital medical expenditure (OR = 5.736–1.829 as compared with the highest quintile of hospital expenditure), good staff attitude (OR = 3.155) as well as good ward environment (OR = 2.361). But patient satisfaction was negatively associated with medical insurance for urban residents and other insurance status (OR = 0.215–0.357 as compared with medical insurance for urban employees). The qualitative analysis showed that patient trust—the most significant predictor of patient satisfaction—is shaped by perceived high quality of service delivery, empathic and caring interpersonal interactions, and a better designed medical insurance that provides stronger financial protection and enables more equitable access to health care.

**Conclusion:**

At the core of high levels of patient dissatisfaction with hospital care is the lack of trust. The current health care system reform in China has yet to address the fundamental problems embedded in the system that caused distrust. A singular focus on doctor-patient inter-personal interactions will not offer a successful solution to the deteriorated patient-provider relationships unless a systems approach to accountability is put into place involving all stakeholders.

## Introduction

The past decade has seen increasing numbers of patient disputes and violence directed towards health care workers in China [1–3]. The Chinese Hospital Management Association (CHMA) estimates that the number of medical disputes has been increasing by 22.9% per year since 2002 [4]. On average, each hospital has to deal with 27 cases of medical violence targeting doctors every year [5]. This has attracted serious concerns from the public and the government alike. Workplace violence has been proved to be negatively associated with job performance [6–9] and quality of life [6, 9–11] of health workers.

Speculations about the causes of the poor patient-provider relationship in China have been made by many commentators: amongst the most blamed factors are poor quality of care (lack of a competent workforce, poor communications, medical errors, lack of respect, poor accessibility); inappropriate financial arrangements (shortage of government investment and profit-driven services); poor health literacy of consumers (poor public understanding, disrespect for knowledge and intellectuals); and inadequate complaint management and legal systems [2, 12–21]. Some empirical studies tried to generate evidence to support such speculations. Wenzhi and colleagues found that insufficient communication, inadequate medical services, and unsatisfactory care outcomes are major predictors of workplace violence [22]. A comparative study of hospital nurses between China and European countries revealed that the poor working environment in China is associated with higher levels of patient dissatisfaction [23].

There is a consensus that patient satisfaction is one of the essential elements of quality care and an indication of good relationships between patients and health workers [24]. The concept of patient satisfaction is multidimensional, and reflects patient perceptions and expectations compared to the actual care they receive [25]. Numerous factors may influence how patients rate their experiences, such as specific individual (met and unmet) needs, care outcomes, prior experience, and comparisons to those of fellow patients [26, 27]. Patient satisfaction reflects the gap between what a patient expects and what he/she receives, in which cultural factors and patient mood may also play a role [28]. Previous studies found that individual demographic characteristics such as age, sex, education and income are associated with patient satisfaction [29, 30]. Determinants of patient satisfaction often go beyond interactions at the individual level between a health worker and a patient [31–33]. Some researchers argue that institutional characteristics of health care organizations, including managerial arrangements, may also be associated with patient satisfaction in their hospital care [34, 35].

A recent Lancet editorial claims that fixing the damaged patient-provider relationships is at the core of health reform in China, and “patient dissatisfaction in China should be considered not as the cause of violence against doctors, but as a symptom of a flawed system that victimises both patients and doctors alike” [12]. Trust is the foundation for all good relationships. Wang and colleagues argued that medical services in China are not expensive compared to other countries [18]. However, the pursuit of financial profits at a systems level in Chinese hospitals [36], irrational inflation of health expenditure and increasing inequalities in health care affordability and health care outcomes [37] have eroded patient-provider trust. The improvement of health workforce competencies, strengthening of law and order and effective management of patient complaints are often proposed as coping strategies for workplace violence [38, 39]. But trust, a critical element in building loyalty and good patient-provider relationships has often been overlooked in Chinese studies [40, 41].

This study, using a mixed methods approach, seeks to explore the role of trust in patient satisfaction with hospital inpatient care, and how patient-provider trust is shaped from the perspectives of both patients and providers at individual, institutional and systemic levels. The findings of such a study can provide evidence and support for the reconstruction of an improved relationship between patients and health care providers.

## Methods

The study was conducted in Heilongjiang province of China. Heilongjiang is located in the northeast of China, with a population of 38.35 million (2013). It had an average GDP of 37,697 Yuan per capita in 2013, ranking in the lowest range of all provinces in China. Patient-doctor relationships in Heilongjiang were reported to be amongst the worst in China [42].

The study involved two stages: an analysis of secondary data drawn from the fifth National Health Service Survey (NHSS, 2013), followed by in-depth interviews to explore views regarding patient-provider relationships from both those who worked in the health industry and those who had no working relationship with the health industry.

### Sampling and data collection

#### Quantitative data

Quantitative data were extracted from the Heilongjiang database of the fifth NHSS (2013). The NHSS is a household survey conducted by the Ministry of Health in China once every five years and systematically gathers the views of consumers about health care services. We sought people who had previously participated in the NHSS through random selection via a multistage stratified cluster sampling method. In Heilongjiang, 30 urban (streets) and 30 rural (townships) local governmental catchments were selected. In each local governmental catchment, two administrative neighborhoods/villages were selected, and eventually, 60 households from each neighborhood/village were invited to participate in the survey [43]. This resulted in a sample of 6,600 households, comprising 18,016 individuals. The Myer’s index and household size indicate a good representative sample compared to the general population structure of Heilongjiang.

Every member of each selected household was interviewed (face-to-face) by a local health worker, gathering information in relation to demographic characteristics (age, sex, education), socio-economic status (employment, income, insurance and living expenses), experience with hospital care (waiting time, ward environment, medical expenditure), overall satisfaction with hospital care, and their level of trust and confidence in care providers. For those under 15 years of age, an adult family member provided a proxy response.

All interviewers participated in intensive training before embarking on fieldwork. To ensure data quality, 5% of the sampled households were randomly selected and revisited by quality control officers. The results showed a high level (95%) of consistency of data. Some 6.7% of all respondents (n = 1214) reported an episode of hospital inpatient care in the previous year. A small number of returned questionnaires contained incomplete data and were therefore excluded from data analysis, which resulted in a final sample size of 1200.

#### Qualitative data

A semi-structured interview guide was developed based on the findings of the quantitative analysis, comprising both pre-determined open questions and prompts encouraging new ideas to be brought up during the interviews. The qualitative study aimed to provide an explanation of the factors associated with patient satisfaction in the quantitative analysis.

The interviews were conducted over the period of 2014 and 2015. Inclusion criteria were based on a balance of participant occupation and their experience with hospital care. Participants were recruited using convenience and snowball sampling. Health workers (n = 7) who were known to the researchers, scholars (n = 5) who worked on patient-provider relationships, and colleagues and neighbors (n = 12) who had experience of hospital care over the past year were the first to be recruited. They were asked to help identify more participants they considered appropriate. These latter recruited participants, in turn, were also encouraged to recommend participants until the sample size was deemed saturated when no new themes emerged. This ended up with a range of 27 health workers (15 health care managers, 12 health professionals) and 35 people (16 officials and managers, 19 consumers) who did not have a working relationship with the health industry.

Researchers and their postgraduate research students from the School of Public Health at Harbin Medical University who have extensive experience in qualitative studies conducted the interviews. Interviews were audio-recorded and transcribed into a word document for further analysis.

### Data analysis

#### Quantitative analysis

Data were analyzed using SPSS 21.0.

Patient satisfaction with hospital inpatient care was rated as “satisfied”, “neutral” and “dissatisfied”. They were recoded into two categories: “satisfied” or “dissatisfied” (including “neutral” and “dissatisfied”) for the purpose of logistic regression modeling.

Independent variables included in the modeling were demographic characteristics (age, sex, education), socio-economic status (employment, income, insurance and living expenses), experience with hospital inpatient care (waiting time, ward environment, disease type and impact of medical expenditure), and level of trust in care providers (Table 1). Annual household income was sorted into five groups using quintile cut-off points derived from the 1200 inpatient sample. The level of trust was originally measured using a 5-point Likert scale (complete distrust; distrust; neither trust nor distrust; trust, complete trust). These were collapsed into two groups: trust (complete trust and trust) and distrust (complete distrust, distrust, neither trust nor distrust) in the modeling.

**Table 1:** Independent variables and coding for regression modeling.

Variable	Characteristic	Coding
**Sex**		
	Female	0
	Male	1
**Age**		
	60 years and older	0
	Below 15 years	1
	15–29 years	1
	30–44 years	1
	45–59 years	1
**Marital Status**		
	Married	1
	Others	0
**Residential location**		
	Rural	1
	Urban	0
**Level of education**		
	College or above	0
	Senior high school	1
	Junior high school	1
	Primary school	1
	None	1
**Employment status**		
	Unemployed	0
	Employed	1
	Retired and student	1
**Annual household income quintile** ^**a**^		
	1	0
	2	1
	3	1
	4	1
	5	1
**Hospital expenditure quintile** ^**b**^		
	1	0
	2	1
	3	1
	4	1
	5	1
**Insurance**		
	Medical Insurance for Urban Employees (MIUE)	0
	Medical Insurance for Urban Residents (MIUR)	1
	New Cooperative Medical Scheme (NCMS)	1
	Others	1
**Waiting time**		
	On/above average	0
	Below average	1
**Level of Trust**		
	Distrust	0
	Trust	1
**Service attitudes of health workers**		
	Poor	0
	Good	1
**Ward environment**		
	Poor	0
	Good	1
**Disease**		
	Cardiovascular	1
	Respiratory	1
	Digestive	1
	Injury and poisoning	1
	Maternal and obstetrics	1
	Others	0

^a^ Quintile 1 is the poorest and quintile 5 the wealthiest

^b^ Quintile 1 is the lowest and quintile 5 the highest

We initially performed Chi-square tests to determine the association between patient satisfaction and each individual variable. We then constructed two models: one including all independent variables (Hosmer-Lemeshow test, χ^2^ = 14.816, P = 0.063) and another one including only those that showed statistical significance (p<0.05) in the Chi-square tests (Hosmer-Lemeshow test, χ^2^ = 5.519, P-value = 0.701). The latter model demonstrated a better fit of model and slightly different Odds Ratios (OR) compared with the first one. As a consequence, we only present the results of the second model.

#### Qualitative analysis

Thematic analysis was performed with the interview data [44]. In the first step, three researchers (LS, YL and QW) read the transcripts repeatedly and verified them with the audio-recordings. They then independently produced descriptive notes, reflecting the underlined meaning of relevant texts, and then shared their notes in meetings. A set of initial codes was generated based on consensus between the three researchers.

In the second step, these codes were collated into potential themes. This involved both inductive and deductive processes. The three researchers developed a conceptual framework illustrating the connections between different themes which was then discussed in a general meeting of the entire project team. The three researchers agreed to place “trust” at the core of the conceptual framework for two reasons. First, the logistic regression model singled out “trust” as the most important predictor of patient satisfaction; second, “trust” emerged as a dominant theme from the interview data set, supported by consensus in the literature that “trust” reflects patients “confidence” and “faith” in health care and care providers. Several trust theories were considered in the development of the conceptual framework [45–47]. Ridd and colleagues distinguished between a generic level of trust in providers *in general* and *personal* trust in a *specific* provider [46]. A patient with a lower level of generic trust in doctors is more likely to make a greater effort to find a doctor who they find empathic to their needs. By contrast, some patients may have blind trust in doctors, but later on lose this trust if an untoward experience occurs. Although competent care is important to patients, a trustful relationship can be jeopardized if doctors fail to recognize the boundaries of their own abilities. An open, honest and trusting relationship may also increase patients’ tolerance of imperfections in health care [47]. Ridd and colleagues proposed to use “knowledge, trust, loyalty and regard” to define the depth of patient-provider relationships [46]. We also considered systemic contexts (resources, finance, policies and regulations) in the conceptual framework because these can shape the expectations of both patients and providers, as well as how each responds to the other’s needs and demands. According to Giddens, trust is directed to the future, which can also be attributed to interactions within and between social groups [45].

In the third step, we checked if the initial codes and the entire data set fit well with the themes and the conceptual framework. Finally, we refined the conceptual framework by relating the analysis to the literature.

### Ethics approval

The study protocol was reviewed and approved by the Research Ethics Committee of Harbin Medical University. In the NHSS, the institutional review board of the Chinese National Bureau of Statistics provided review and ethics approval of the survey, all respondents were read a statement that explained the purpose of the survey and gave consent to continue. We obtained written informed consent from our interviewees.

## Results

### Characteristics of respondents and patient satisfaction

More than half of all respondents (52.6%) were female, 38.5% were 60 years or older. The majority of respondents were married (82.5%) and had no tertiary education (97.2%) at the time of the survey.

Overall, about 24% of the respondents were dissatisfied with their hospital inpatient care. Chi-square tests revealed that patient satisfaction was associated with age, residential location, medical expenditure, insurance status, level of trust, service attitudes of health workers, and ward environment (Table 2). Older people, rural residents, patients with a smaller hospital bill, members of the medical insurance scheme for urban employees, those who had a higher level of trust in health workers, and those who felt happy with the ward environment and staff attitudes were more likely to express satisfaction towards hospital inpatient care (Table 2).

**Table 2:** Characteristics of respondents and overall satisfaction towards hospital inpatient care (n = 1200).

Characteristic	n (%)	Satisfied n (%)	Dissatisfied n (%)	χ ^2^	*p*-value
**Sex**				0.078	0.781
Male	569 (47.4)	428 (75.2)	141 (24.8)		
Female	631 (52.6)	479 (75.9)	152 (24.1)		
**Age**				14.034	0.007
<15	89 (7.4)	58 (65.2)	31 (34.8)		
15–29	75 (6.3)	57 (76.0)	18 (24.0)		
30–44	160 (13.3)	118 (73.8)	42 (26.3)		
45–59	414 (34.5)	301 (72.7)	113 (27.3)		
≥60	462 (38.5)	373 (80.7)	89 (19.3)		
**Marital Status**				1.866	0.172
Married	990 (82.5)	756 (76.4)	234 (23.6)		
Others	210 (17.5)	151 (71.9)	59 (28.1)		
**Residential location**				12.468	0.000
Rural	1019(84.9)	789 (77.4)	230 (22.6)		
Urban	181 (15.1)	118 (65.2)	63 (34.8)		
**Level of education**				6.274	0.180
None	232 (19.3)	176 (75.9)	56 (24.1)		
Primary	482 (40.2)	376 (78.0)	106 (22.0)		
Junior high school	388 (32.3)	282 (72.7)	106 (27.3)		
Senior high school	65 (5.4)	45 (69.2)	20 (30.8)		
College or above	33 (2.8)	28 (84.8)	5 (15.2)		
**Employment status**				0.663	0.718
Employed	653 (54.4)	498 (76.3)	155 (23.7)		
Unemployed	320 (26.7)	242 (75.6)	78 (24.4)		
Retired and student	227 (18.9)	167 (73.6)	60 (26.4)		
**Annual household income quintile** ^**a**^				5.450	0.244
1	259 (21.6)	192 (74.1)	67 (25.9)		
2	223 (18.6)	162 (72.6)	61 (27.4)		
3	255 (21.3)	197 (77.3)	58 (22.7)		
4	326 (27.2)	243 (74.5)	83 (25.5)		
5	137 (11.4)	113 (82.5)	24 (17.5)		
**Hospital expenditure quintile** ^**b**^				37.107	0.000
1	246 (20.5)	214 (87.0)	32 (13.0)		
2	256 (21.3)	205 (80.1)	51 (19.9)		
3	220 (18.3)	160 (72.7)	60 (27.3)		
4	238 (19.8)	172 (72.3)	66 (27.7)		
5	240 (20.0)	156 (65.0)	84 (35.0)		
**Insurance status**				32.884	0.000
Medical Insurance for Urban Employees	107 (8.9)	84 (78.5)	23 (21.5)		
Medical Insurance for Urban Residents	45 (3.8)	20 (44.4)	25 (55.6)		
New Cooperative Medical Scheme	934 (77.8)	728 (77.9)	206 (22.1)		
Others	114 (9.5)	75 (65.8)	39 (34.2)		
**Waiting time**				2.648	0.104
Below average	1157(96.4)	879 (76.0)	278 (24.0)		
On/above average	43 (3.6)	28 (65.1)	15 (34.9)		
**Level of Trust**				219.795	0.000
Trust	1112(92.7)	898 (80.8)	214 (19.2)		
Distrust	88 (7.3)	9 (10.2)	79 (89.8)		
**Service attitudes of health workers**				206.062	0.000
Good	1020(85.8)	853 (82.8)	177 (17.2)		
Poor	170 (14.2)	54 (31.8)	116 (68.2)		
**Ward environment**				106.714	0.000
Good	821 (68.4)	692 (84.3)	129 (15.7)		
Poor	379 (31.6)	215 (56.7)	164 (43.3)		
**Disease**				6.663	0.247
Cardiovascular	470(39.4)	357(76.0)	113(24.0)		
Respiratory	154(12.8)	125(81.2)	29(18.8)		
Digestive	124(10.3)	96(77.4)	28(9.6)		
Injury and poisoning	83(6.9)	57(68.7)	26(31.3)		
Maternal and obstetrics	69(5.8)	54(78.3)	15(21.7)		
Others	300(25.0)	218(72.7)	82(27.3)		

^a^ Quintile 1 is the poorest and quintile 5 the wealthiest

^b^ Quintile 1 is the lowest and quintile 5 the highest

### Logistic regression model

Those independent variables with statistical significance in the Chi-square tests were entered into the logistic regression model. “Trust” appeared to be the most significant predictor of patient satisfaction, followed by level of hospital medical expenditure, medical insurance status, health worker empathy, and ward environment (Table 3). The odds of being satisfied with hospital inpatient care by those who trusted health workers was almost fifteen times (OR = 14.995) of those who had little faith in health workers. Compared with the respondents covered by the insurance scheme for urban employees, members of the medical insurance scheme for urban residents were less likely to be satisfied with their hospital inpatient care (OR = 0.215). The gap in the odds of being satisfied with hospital inpatient care between those with different hospital bills was up to 5.7 times (OR ranging from 1.829 to 5.736). Empathic staff (OR = 3.155) and ward environment (OR = 2.361) were also significant predictors of patient satisfaction. We noted that individual demographic characteristics (age and residential location) became insignificant in the logistic regression model.

**Table 3:** Logistic regression analysis on inpatients’ overall satisfaction.

Variables	Walds	*p*	OR	95%CI	
**Age**					
<15	2.338	0.126	0.614	0.329	1.147
15–29	0.423	0.515	0.793	0.394	1.595
30–44	1.487	0.223	0.727	0.436	1.213
45–59	3.893	0.048	0.686	0.471	0.998
60 years and older (reference)					
**Residential location**					
Rural	1.153	0.283	1.586	0.684	3.679
Urban(reference)					
**Insurance status** ^**a**^					
MIUR	11.264	0.001	0.215	0.088	0.528
NCMS	1.996	0.158	0.492	0.184	1.316
Others	4.144	0.042	0.357	0.133	0.962
MIUE (reference)					
**Hospital expenditure quintile** ^**b**^					
1	38.534	0.000	5.736	3.304	9.957
2	19.884	0.000	3.026	1.860	4.922
3	8.444	0.004	2.024	1.258	3.257
4	6.920	0.009	1.829	1.166	2.867
5 (reference)					
**Level of Trust**					
Trust	45.426	0.000	14.995	6.823	32.953
Distrust (reference)					
**Service attitudes of health workers**					
Good	21.112	0.000	3.155	1.933	5.151
Poor (reference)					
**Ward environment**					
Good	20.637	0.000	2.361	1.630	3.421
Poor (reference)					
**Constants**	35.939	0.000	0.048		

^a^ MIUE—Medical Insurance for Urban Employees; MIUR—Medical Insurance for Urban Residents; NCMS—Rural New Cooperative Medical Scheme.

^b^ Quintile 1 is the lowest and quintile 5 the highest

### Perceived reasons for dissatisfaction with hospital inpatient care

**Fig 1:**
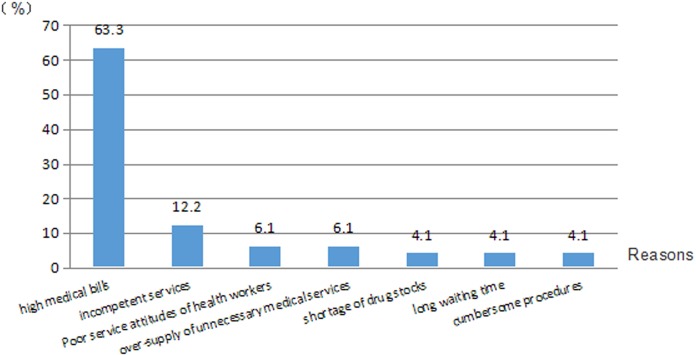
Reasons for dissatisfaction with hospital inpatient care.

Among those who expressed dissatisfaction with hospital inpatient care, 63% attributed it to high medical bills and a further 12% complained about incompetent healthcare delivery (Fig 1). A lack of empathy demonstrated by health workers (6.1%), over-supply of unnecessary medical services (6.1%), drug unavailability (4.1%), excessive waiting times (4.1%) and cumbersome procedures (4.1%) were reported by some respondents as reasons for dissatisfaction.

### Factors undermining trust between patients and providers

The interviewees regard ethical consistently overwhelmingly as the foundation for patient-provider relationships. Patient trust was singled out as the core for building good patient-provider relationships. Additionally, they commonly agree that patient-provider relationships in China are distorted in favor of economic gain.

**Fig 2:**
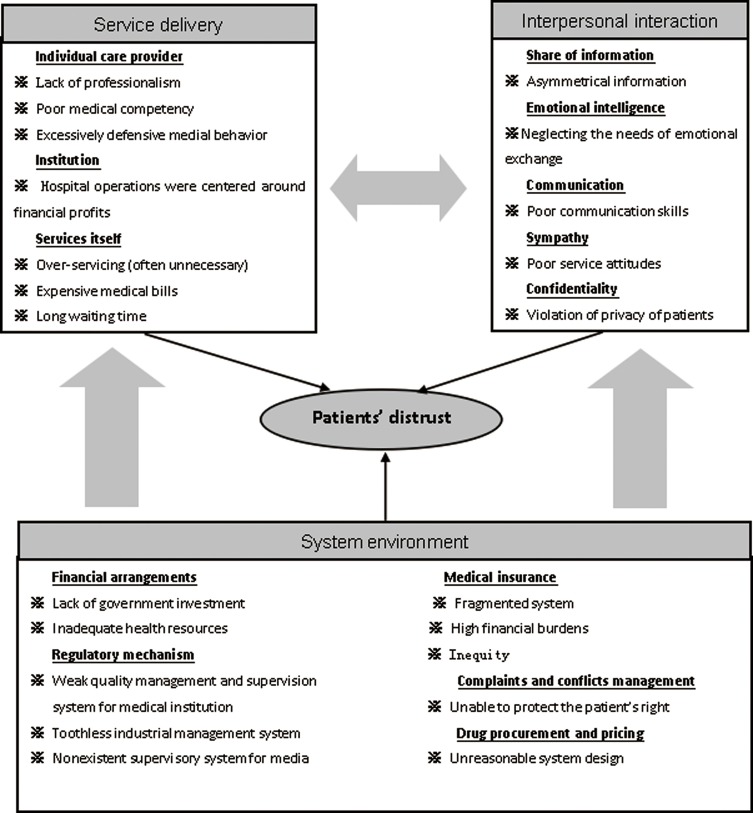
Conceptual framework of patients’ distrust in care providers.

Three broad themes emerged as key factors that undermined the development and maintenance of trust between patients and doctors: service delivery, empathic and caring interpersonal interactions, and system environment (Fig 2). *Service delivery* describes what and how services are provided from the consumer’s perspective. Assurance of optimal safe care that meets the needs of patients may enhance the trust of providers. *Interpersonal interaction* indicates how patients and care providers exchange information and ideas and make decisions in a manner that the health consumer feels the provider understands their perspective, and has a shared investment in the outcome. While “*service delivery*” is expected to be bound with scientific evidence (what should be delivered and how), *interpersonal interaction* is sensitive to individual social, cultural, and economic circumstances. Sometimes, interpersonal interaction may even go beyond the scope of services. We posit that “*service delivery*” and “*interpersonal interaction*” are shaped by “*system environment*”. If a certain medicine is deemed necessary but unaffordable by a patient (system environment), the doctor has to find an alternative solution (service delivery) and communicates with the patient in a different way (interpersonal interaction).

*Service delivery* is embedded in the individual care provider, institution, and the service itself. Patients tend to assess service delivery through tangible elements: (1) At the individual care provider level, interviewees expressed their view that a lack of professionalism would jeopardize trust in providers, in terms of medical officers prescribing excessive and unnecessary prescriptions and high-technology examinations for their own financial interest, rather than for the patient’s benefit. A failure of medical competency was considered the root cause of misdiagnosis, unnecessary treatment and iatrogenic harm. *Patient distrust*, on the other hand, further exacerbated defensive medical practices. (2) At the institutional level, hospital operations are fixed to ensure financial solvency, competing for market advantage over volume of services. Priorities are given to high-quality medical resources (highly visible and expensive) instead of high quality services (less visible). (3) The service itself can be described in terms of appropriateness, cost and waiting time. Over-servicing (often unnecessary), exorbitant medical bills, and long waiting times are antithetical to trust in care providers.

*Interpersonal interaction* encompasses five main elements: (1) Sharing information—Medical information was usually collected and recorded in a way that was difficult for patients to comprehend; (2) Emotional intelligence—Health providers were trained to deliver “hard scientific” services, often neglecting the impact that a lack of empathy produces; (3) Communication—Poor communication skills of health providers jeopardized consumer confidence; (4) Sympathy—Poor service attitudes of health workers exacerbated patient distrust; and (5) Confidentiality—Violation of patient information privacy fueled patient distrust.

The *system environment*, if deviating from the shared values of the society, may contribute to a sense of inequity. This compounds the development of distrust in care providers, and shapes the ways in which patients and care providers interact. Five elements emerged as important aspects of the system environment: (1) Financial arrangements—Lack of government investment forces hospitals to maximize financial security by pursuing profits from user charges. Meanwhile, primary care institutions are inadequately resourced, pushing more patients to seek care from hospitals. (2) Medical insurance–Multiple insurance schemes exist for different groups of consumers. This fragmented system lacks any ability to influence provider behaviors. With limited insurance funds available, the over-provision of services and rising medical expenditure, financial burdens on consumers remain high by the imposition of considerable co-payments, sometimes leading to intergenerational poverty. (3) Regulatory mechanisms—Government capacity in regulating the hospital sector is weak because it has little financial impact. The industry itself is neither willing nor able to effectively achieve self-regulation. The public media were viewed as irresponsible and misleading, often fueling conflicts between patients and hospitals. (4) Complaints and conflict management–It was widely accepted that current complaints and conflict resolution systems have failed to protect and enhance the rights and interests of patients. Cumbersome medical dispute procedures, excessive waiting times and poor conflict resolution outcomes encourage some patients to resort to undesirable and even violent measures to seek express dissatisfaction or achieve compensation. (5) Drug procurement and pricing–Limited drug availability (restricted by the government) in primary care institutions and higher drug prices in hospitals contribute to increased financial burden associated with medicines for some patients.

## Discussion

The level of patient satisfaction with hospital care is low in Heilongjiang: more than 20% of respondents were dissatisfied with their hospital care, far higher than the national average [48].

Trust was found to be the most important factor contributing to patient satisfaction. The odds of being satisfied with hospital inpatient care by those who trust care providers is about fifteen times (OR = 14.995) of those who do not trust care providers. Such a high OR is extraordinary compared with the findings of similar studies in other countries. For instance, Footman and López-Cevallos found only a differential of one time in the odds of being satisfied between people who trusted and distrusted [49, 50].

A wide range of factors complicates patient’s distrust in health providers in China, hence, reinstating and maintaining patient trust is a great challenge in China. Problems in relation to service delivery and interpersonal interaction and their associations with patient trust have been studied extensively. Similar to findings from other studies [51, 52], our logistic model shows that empathic staff attitudes (OR = 3.155) and ward environment (OR = 2.361) are associated with high levels of patient satisfaction. Cai and Zhang also found that poor facilities, cumbersome procedures, uncaring attitudes and inflated medical bills are among the major complaints of hospital inpatients [53]. According to Hsiao and Zhong, more than one third of pharmaceutical expenditure is associated with over-prescription [54, 55]. It is also common for medical staff to accept red envelopes (cash bribes) from patients [1]. Such a monetary relationship imposes a serious threat to the development of trust between patients and providers.

In China, doctors often become a scapegoat for poor patient satisfaction and in medical conflicts. However, the reasons behind poor services and interpersonal interactions are much more complicated than many people think. Most Chinese medical curricular treat medical practice as a hard science, with limited attention to humanistic aspects such as ethics and communication skills [56]. The number of medical staff per thousand population in China is very low compared with developed nations [57], contributing to high workloads and long working hours (54.06 ±10.76 hours a week) [58].

The individual healthcare financial impact remains an important determinant for people making medical decisions. In our study, we found that making individual healthcare costs more affordable can make a difference of up to 5.7 times in the odds of patients being satisfied with hospital care. An opinion piece recently published in the Lancet argued that increased out-of-pocket health care costs are a major cause of patient dissatisfaction [19, 59]. Indeed, on average, health expenditure per capita increased more than four times in China from the year 2000 to 2010 [60].

Researchers believe that such a rapid rise in health expenditure is a result of not only economic and technology progress, but also inadequate policy and system design [19, 54]. A shortage of government investment is evident, which has resulted in hospitals’ complete dependence on user charges. Health expenditure in China accounts for only 3% of the total world health expenditure, yet its population accounts for 20% of the world’s total [61]. Fee-for-service payment mechanisms are an irresistible incentive for hospitals and doctors to increase volumes of services [62]. Such perverse incentives are particularly strong when doctors feel dissatisfied with their income: a survey of 720 doctors in three provinces revealed that only 8% felt satisfied with their income [63]. Indeed, when compared to their international counterparts, medical officer remuneration in China is modest [19], which contributes to patient interests being wedged between economic temptation and professional ethics [1, 19].

Despite near universal coverage of medical insurance, urgent attention needs to be paid to inequity across insurance schemes. This study revealed a disparity in patient satisfaction between members of different insurance schemes. Those with medical insurance for urban residents were less likely (OR = 0.215) to be satisfied with hospital care compared with their counterparts with medical insurance for urban employees. The benefits for MIUR members are limited by its low financing level [64, 65]. The 2008 NHSS reported a 49.3% reimbursement rate for MIUR members, compared with 63.2% for MIUE members [66]. The actual gap in reimbursement rate, according to a study, could be as high as 1.5 times between MIUR and MIUE [67]. This is concerning because reducing inequity is one of the most important considerations in the design of medical insurance schemes.

The reputation of the hospital sector in China is impaired, partly because of the sensationalized depiction of medical incidents and conflicts by the public media. Some researchers have realized that these reports are misleading: blaming hospitals for poor service delivery and excessively high and unnecessarily high medical costs for consumers, but ignoring the causative structural imperatives [68]. The widespread atmosphere of suspicion fostered by the media perversely encourages defensive practice: the very issue the media decries. In some cases, patients with serious illnesses are refused admission to hospitals. Even when patients are treated, doctors prefer to order excessive diagnostic tests purely for legal defensibility. A vicious circle of distrust exists in China [69, 70].

## Conclusion and policy recommendations

Patient satisfaction with hospital inpatient care is low in Heilongjiang in China. The fundamental problem undermining the poor rating of patient experiences is the lack of trust. In addition, high out-of-pocket payments, inequality of insurance entitlements, and perceived poor quality of services also contribute to patient dissatisfaction with hospital inpatient care. Therefore, reform the current medical insurance system that targeting at providing stronger financial support to reduce patients’OOP payment and designing more equitable benefit package across different medical insurance schemes as well as enhancing service quality will be the key to address the problems of distrust.

This is one of the rare studies exploring the role of trust in patient rating of their hospital experiences from individual, institutional and systemic levels. Factors associated with service delivery, interpersonal interactions, and system environment intertwine to shape trust in care providers. The findings of this study indicate that a singular focus on doctor-patient inter-personal interactions will not offer a successful solution to the deteriorated patient-provider relationships unless a systems approach to accountability is put into place involving all stakeholders including government, health professionals, health insurance, medical education, and the public. The current health care system reform in China is yet to address the fundamental problems embedded in the health system that cause high levels of patient dissatisfaction and distrust.

This study offers some policy implications for China’s health reforms. At the individual level, medical standards and professional ethics should be strengthened, reviewed and enforced. Professional education should place more emphasis on empathic communication skills and patient-centered care. At the institutional level, priority should be given to patient safety and quality of care in all aspects of healthcare provision. This includes, but is not limited to, resource support, appropriate workloads, appropriate remuneration based on skills and accountability, and improved work environment. At the system level, policy alignment is critical. With increased government investment, improving benefits and equity of medical insurance schemes should become a focus in health reform. Payment mechanisms for healthcare must encourage prudence and value for money. Actions need to be taken by the government, professional bodies, consumer organizations, and the public media to rebuild patient confidence and trust in health services. Meanwhile, it is important to improve the health literacy of consumers so that they can have a better understanding and clearer expectations of health care services.

## Supporting Information

S1 Appendix The fifth National Health Service Survey—Questionnaire. (DOC)

S2 Appendix Factors undermining trust between patients and providers—Interview guide. (DOC)
