# Supplementary material for: Patient Satisfaction with Hospital Inpatient Care: Effects of Trust, Medical Insurance and Perceived Quality of Care
Source: PLoS One. 2016 Oct 18;11(10):e0164366. doi: 10.1371/journal.pone.0164366 (PMC5068749; doi:10.1371/journal.pone.0164366)
Supplement: S1 Appendix — (DOC) [file pone.0164366.s001.doc]

|  |  | **表 号：国卫调1 表**  **制定机关：国家卫生计生委**  **批准机关：国家统计局**  **批准文号：国统制[2013]65号**  **有效期至：2013年12月** |
| --- | --- | --- |
|  |
|  |

**家庭健康询问调查表**

**家庭地址：__________县（市/区）_________乡镇（街道）_________村（居委会） ___________________________（详细地址）**

**户主姓名：_____________ 联系电话：**

**县（市/区）行政区划代码□□□□□□ 乡镇（街道）代码□□□ 村（居委会）代码□□□**

**住户代码□□**

**调查开始时间: 2013年 月**   **日 时 分**

**调查完成时间: 2013年 月**   **日 时 分 调查员（签名）：**

**核实日期： 2013年 月**   **日** **调查指导员（签名）：**

**调查员入户致辞**

**尊敬的居民：**

**您好！我们是第五次国家卫生服务调查的调查员。本次调查由国家卫生管理部门统一组织，调查内容经过了国家统计局的批准。国家卫生服务调查的主要目的是要了解居民健康状况和医疗卫生服务利用情况，为国家制定卫生政策，改善居民健康水平提供信息。所有调查内容仅用于统计分析，我们将按照《中华人民共和国统计法》相关条款要求，对您及家人的信息予以保密。希望您能如实回答下面的问题，非常感谢您的支持与配合！**

**“统计调查中获得的能够识别或者推断单个统计调查对象身份的资料，任何单位和个人不得对外提供、泄露，不得用于统计以外的目的。”**

**《中华人民共和国统计法》（第三章第二十五条）**

调查表清单

表1. 家庭一般情况调查表

表2. 家庭成员个人情况调查表

表3. 调查前两周内病伤情况调查表

表4. 调查前一年内住院情况调查表

表5. 5岁以下儿童调查表

表6. 15-64岁妇女调查表

**表1. 家庭一般情况调查表**

**本表由被调查户中最熟悉家庭情况的人回答**

| 序号 | 问题及选项 | 回答 |
| --- | --- | --- |
|  | 您家户籍人口数？（户口本上的人口） |  |
|  | 户籍人口中，近6个月内有几人在家里居住？ |  |
|  | 近6个月内住在您家里，但户口不在您家的人数？（包括亲友、保姆等） |  |
|  | （农村地区询问）户籍人口中，近6个月内有几人在县外务工？（包括随行人员，如配偶、孩子和父母等） |  |
|  | 离您家最近的医疗机构有多少公里： ⑴不足1公里 ⑵ 1- ⑶ 2- ⑷ 3- ⑸ 4- ⑹ 5公里及以上 |  |
|  | 从您家到最近医疗机构需要多少分钟？（以步行或搭乘交通工具等容易获得的最快方式） |  |
|  | 对于一般性疾病，您家里人通常去哪类医疗机构就医：  ⑴诊所/村卫生室 ⑵社区卫生服务站 ⑶卫生院 ⑷社区卫生服务中心 ⑸综合医院 ⑹中医医院 ⑺其它 |  |
|  | 与5年前相比,您家人在看病方便程度方面有什么变化： ⑴大幅改善 ⑵略有改善 ⑶没有变化 ⑷略有恶化 ⑸大幅恶化 |  |
|  | 与5年前相比,您家人在看病花费方面有什么变化： ⑴大幅下降 ⑵略有下降 ⑶没有变化 ⑷略有增加 ⑸大幅增加 |  |
|  | 您认为医生和患者最类似于下列哪种关系：  ⑴父母与子女 ⑵师生 ⑶朋友 ⑷工作伙伴 ⑸战友 ⑹上下级 ⑺买卖服务 ⑻其他 |  |
|  | 您家烹饪最常使用的燃料是： ⑴电　 ⑵煤气/天然气/液化石油气 ⑶沼气 ⑷煤油 ⑸煤炭 ⑹柴草 ⑺其它 |  |
|  | 您家饮用水类型： ⑴自来水 ⑵手压机井水 ⑶受保护的水井 ⑷雨水收集 ⑸受保护的泉水  ⑹未受保护的井水 ⑺未受保护的泉水 ⑻卡车或手推车送水 ⑼地表水 ⑽其它 |  |
|  | 您家厕所类型： ⑴完整下水道水冲式 ⑵粪尿分集式 ⑶三联沼气 ⑷双瓮漏斗式 ⑸三格化粪池  ⑹双坑交替式 ⑺通风改良式 ⑻阁楼式 ⑼深坑防冻式 ⑽有盖板的坑式厕所  ⑾无盖板的坑式厕所 ⑿粪桶 ⒀无设施或灌木丛或田间 ⒁其它 |  |
|  | 您家住房类型是： ⑴楼房 ⑵砖瓦平房 ⑶土坯平房 ⑷其它 |  |
|  | 您家生活住房建筑面积约多少平方米? |  |
|  | 您家前一年总收入约为多少元？（城镇居民家庭为可支配收入，农村居民家庭为纯收入） |  |
|  | 您家前一年生活消费性支出共为多少元？ |  |
|  | 其中： 食品支出多少元？ |  |
|  | 衣着及日用品支出多少元？ |  |
|  | 交通、通信支出多少元？ |  |
|  | 住房、水电及燃料支出多少元？ |  |
|  | 教育支出多少元？ |  |
|  | 文化及娱乐支出多少元？ |  |
|  | 药品、医疗服务及用品支出多少元？ |  |
|  | 其它支出多少元？ |  |
|  | 您家是否被列为本地的贫困户？ ⑴是 ⑵否 |  |
|  | 您家是否被列为本地的低保户？ ⑴是 ⑵否 |  |
|  | 若是贫困户或低保户，您认为导致经济困难的最主要原因是什么：  ⑴劳动力人口少 ⑵自然条件差或灾害 ⑶因疾病损伤影响劳动能力 ⑷因治疗疾病  ⑸失业或无业 ⑹人为因素 ⑺其它 |  |

**表2. 家庭成员个人情况调查表**

| **A. 个人基本情况** | |  |  |  |  |  |  |
| --- | --- | --- | --- | --- | --- | --- | --- |
| **被调查成员代码 （01为户主，其他按调查顺序自行编码，成员代码一旦确定，不能更改）** | | 01  户主 | 02 | 03 | 04 | 05 | 06 |
|  | 成员姓名： （01为实际户主） |  |  |  |  |  |  |
|  | 该成员与户主的关系：  ⑴户主本人 ⑵配偶 ⑶子女　 ⑷女婿/儿媳  ⑸父母 ⑹岳父母/公婆 ⑺祖父母 ⑻孙子女  ⑼兄弟/姐妹 ⑽家政服务人员 ⑾其他 |  |  |  |  |  |  |
|  | 下列调查问题由谁回答**（调查员判断）**： ⑴自己回答  ⑵他人代答 |  |  |  |  |  |  |
|  | 户口登记地： ⑴本县/区 ⑵本省外县/区  ⑶外省 ⑷户口待定 |  |  |  |  |  |  |
|  | 户口性质： ⑴农业 ⑵非农业 |  |  |  |  |  |  |
|  | 性别： ⑴男 ⑵女 |  |  |  |  |  |  |
|  | 民族： ⑴汉族 ⑵壮族 ⑶回族 ⑷维族 ⑸蒙族  ⑹藏族 ⑺满族 ⑻苗族 ⑼其它 |  |  |  |  |  |  |
|  | 出生日期： 年份 **（填写4位数字，如：1998）** |  |  |  |  |  |  |
|  | 月份 **（填写2位数字，如：07）** |  |  |  |  |  |  |
|  | 您的身高是多少（厘米）？ |  |  |  |  |  |  |
|  | 您的体重是多少（公斤）？ |  |  |  |  |  |  |
| 被调查成员代码 | | 01 | 02 | 03 | 04 | 05 | 06 |
|  | 您是否参加了城镇职工基本医疗保险？ ⑴是 ⑵否 |  |  |  |  |  |  |
|  | 您是否参加了城镇居民基本医疗保险？ ⑴是 ⑵否 |  |  |  |  |  |  |
|  | 您是否参加了新型农村合作医疗？ ⑴是 ⑵否 |  |  |  |  |  |  |
|  | 您是否参加了城乡居民合作医疗保险？ ⑴是 ⑵否 |  |  |  |  |  |  |
|  | 您是否购买了商业医疗保险？ ⑴是 ⑵否 |  |  |  |  |  |  |
|  | 您是否参加了其它医疗保险？ ⑴是 ⑵否 |  |  |  |  |  |  |
|  | 您是否为政府的医疗救助对象？ ⑴是 ⑵否 ⑶不知道 |  |  |  |  |  |  |
| **继续询问15岁及以上人口（1998年8月15日以前出生）,15岁以下人口转问表3** | | | | | | | |
|  | 婚姻状况： ⑴未婚 ⑵已婚 ⑶丧偶  ⑷离婚 ⑸其它 |  |  |  |  |  |  |
|  | 文化程度： ⑴没上过学 ⑵小学 ⑶初中  ⑷高中 ⑸技工学校 ⑹中专(中技)  ⑺大专 ⑻本科及以上 |  |  |  |  |  |  |
|  | 就业状况： ⑴在业(包括灵活就业） ⑵离退休  ⑶在校学生 ⑷失业 ⑸无业 |  |  |  |  |  |  |
|  | 职业类型（询问在业和离退休人员）：  ⑴机关、企事业单位负责人 ⑵专业技术人员  ⑶办事人员和有关人员 ⑷商业/服务业人员  ⑸农林牧渔水利业生产人员 ⑹生产运输设备操作人员  ⑺军人 ⑻其它 |  |  |  |  |  |  |
| **B. 身体功能** | |  |  |  |  |  |  |
| 被调查成员代码 | | 01 | 02 | 03 | 04 | 05 | 06 |
|  | 今天您在行动方面：  ⑴四处走动,无任何困难 ⑵行动有些不便  ⑶不能下床活动 |  |  |  |  |  |  |
|  | 今天您自我照顾（盥洗、穿衣上厕所等）方面：  ⑴无任何问题 ⑵有些问题  ⑶无法自己盥洗或穿衣服 |  |  |  |  |  |  |
|  | 今天您从事平常活动（工作、读书或做家务）方面：  ⑴从事日常活动无任何问题 ⑵有些问题  ⑶无法从事日常活动 |  |  |  |  |  |  |
|  | 今天您身体疼痛或不舒服方面：  ⑴无任何疼痛或不舒服 ⑵自觉有中度疼痛或不舒服  ⑶自觉极度疼痛或不舒服 |  |  |  |  |  |  |
|  | 今天您在焦虑或抑郁方面：  ⑴不觉得焦虑或抑郁 ⑵自觉中度焦虑或抑郁  ⑶自觉极度焦虑或抑郁 |  |  |  |  |  |  |
|  | 请您说出最能代表您今天健康状况好坏的那个分值  ├---┼—-┼—-┼—-┼—-┼—-┼—-┼—-┼—-┼—-┤  0 10 20 　30 　40 　50 60 70 80 90 100  最差健康状况 最好健康状况 |  |  |  |  |  |  |
| **C. 健康行为** | |  |  |  |  |  |  |
| 被调查成员代码 | | 01 | 02 | 03 | 04 | 05 | 06 |
|  | 您现在的吸烟状况：  ⑴每天吸 ⑵非每天吸 ⑶不吸***(跳问60)*** |  |  |  |  |  |  |
|  | 您开始吸烟的年龄（岁）？ |  |  |  |  |  |  |
|  | 近一周内，您平均每天吸多少支烟（支）？***(跳问61)*** |  |  |  |  |  |  |
|  | 您过去的吸烟状况： ⑴每天吸 ⑵非每天吸 ⑶不吸 |  |  |  |  |  |  |
|  | 近12个月内，您喝过酒吗： ⑴是 ⑵否***（跳问64）*** |  |  |  |  |  |  |
|  | 您的饮酒频率有多大：  ⑴每周至少3次 ⑵每周1-2次 ⑶每周不到1次 |  |  |  |  |  |  |
|  | 您平均每次饮酒的量相当于多少饮酒单位（标准饮酒单位）？  **(由调查员换算)**  （1两40度及以上白酒=2； 1两40度以下白酒=1.5；  1斤葡萄酒=5；1瓶啤酒=2； 1听啤酒=1； 1斤黄酒=6.5) |  |  |  |  |  |  |
|  | 近6个月内，您平均每周体育锻炼几次：  ⑴ 6次及以上 ⑵ 3-5次 ⑶ 1-2次  ⑷不到1次 ⑸ 从不锻炼***（跳问67）*** |  |  |  |  |  |  |
|  | 您平均每次锻炼的强度是多大（自我呼吸、心跳加快的感觉）：  ⑴轻度 ⑵中度 ⑶重度 |  |  |  |  |  |  |
|  | 您平均每次锻炼多长时间（分钟）？ |  |  |  |  |  |  |
| 被调查成员代码 | | 01 | 02 | 03 | 04 | 05 | 06 |
|  | 您是否有健康档案?  ⑴是 ⑵否 ⑶不知道 |  |  |  |  |  |  |
|  | 近12月内，您是否进行过健康体检？（不包括因病做的检查）  ⑴是 ⑵否 |  |  |  |  |  |  |
|  | 您平均每天刷几次牙：  ⑴ 2次及以上 ⑵ 1次 ⑶不到1次 ⑷不刷牙 |  |  |  |  |  |  |
| **D. 慢性疾病** | |  |  |  |  |  |  |
|  | 您是否被医生确诊患有高血压病？  ⑴是 ⑵否***（跳问75）*** |  |  |  |  |  |  |
|  | 您目前服用降血压药物的频率为：  ⑴按医嘱每天服用 ⑵偶尔或必要时服用 ⑶从不服用 |  |  |  |  |  |  |
|  | 您最近一次测量血压的时间：  ⑴一周内 ⑵一个月内 ⑶三个月内  ⑷半年内 ⑸半年以前 |  |  |  |  |  |  |
|  | 您目前的血压是否正常？ ⑴是 ⑵否 ⑶不清楚 |  |  |  |  |  |  |
|  | 近三个月内，是否有医务人员对您进行高血压病防治指导？  ⑴是 ⑵否 |  |  |  |  |  |  |
|  | 您是否被医生确诊患有糖尿病？  ⑴是 ⑵否***（跳问80）*** |  |  |  |  |  |  |
|  | 您目前使用降血糖药物的频率为：  ⑴按医嘱每天使用 ⑵偶尔或必要时使用  ⑶从不使用***（跳问78）*** |  |  |  |  |  |  |
| 被调查成员代码 | | 01 | 02 | 03 | 04 | 05 | 06 |
|  | 您目前如何使用降血糖药物：  ⑴口服 ⑵注射 ⑶二者都用 |  |  |  |  |  |  |
|  | 您最近一次测量血糖的时间（包括医疗机构测量和自我测量）：  ⑴一月内 ⑵三个月内 ⑶半年内 ⑷半年以前 |  |  |  |  |  |  |
|  | 您目前的血糖值是否正常？  ⑴是 ⑵否 ⑶不清楚 |  |  |  |  |  |  |
|  | 近6个月内，您是否患有被医生确诊的其它慢性疾病? *  ⑴是 ⑵否***（跳问84）*** |  |  |  |  |  |  |
|  | 第一种慢性疾病 （疾病名称）  （如果有多种慢性病，按患病严重程度由高到低依次填写） |  |  |  |  |  |  |
| 81A | 查填第一种疾病代码 |  |  |  |  |  |  |
|  | 第二种慢性疾病 （疾病名称） |  |  |  |  |  |  |
| 82A | 查填第二种疾病代码 |  |  |  |  |  |  |
|  | 第三种疾病 （疾病名称） |  |  |  |  |  |  |
| 83A | 填查第三种疾病代码 |  |  |  |  |  |  |

***注：慢性病指符合下列情况之一者：**

①调查前半年内，经过医务人员明确诊断的慢性病；

②调查半年以前患有医生诊断的慢性病，在调查前半年内时有发作并采取了治疗措施如服药、理疗，或者一直在治疗以控制慢性病的发作等。

| **E. 60岁及以上人口健康（询问1953年8月15日以前出生人口，其他人转问表3）** | | | | | | | |
| --- | --- | --- | --- | --- | --- | --- | --- |
| 被调查成员代码 | | 01 | 02 | 03 | 04 | 05 | 06 |
|  | 您最主要经济来源是： ⑴自己或配偶 ⑵子女 ⑶孙子女  ⑷亲戚 ⑸朋友 ⑹社会救济 ⑺其它 |  |  |  |  |  |  |
|  | 近6个月内，您在行走方面属于下列哪种情况：  ⑴长期卧床，有人帮助才能坐起 ⑵没人帮助，不能行走  ⑶没人帮助，不能独自出门上街 ⑷行走自如 |  |  |  |  |  |  |
|  | 近6个月内，您在听力方面属于下列哪种情况：  ⑴很难听清楚 ⑵需要别人提高声音 ⑶能听清楚 |  |  |  |  |  |  |
|  | 近6个月内，您说话是否有困难？ ⑴是 ⑵否 |  |  |  |  |  |  |
|  | 近6个月内，您辨认出20米外熟人的困难程度： (戴眼镜者，回答戴眼镜时的情况)  ⑴没有或轻度困难 ⑵自觉中度困难 ⑶自觉极度困难 |  |  |  |  |  |  |
|  | 近1个月内，您的生活起居是否需要别人照顾？ ⑴是 ⑵否 |  |  |  |  |  |  |
|  | 需要照顾时，主要由谁来提供帮助：  ⑴配偶 ⑵子女 ⑶孙子女 ⑷兄弟姐妹 ⑸亲戚 ⑹邻居 ⑺保姆 ⑻社区 ⑼其他 ⑽没人帮助 |  |  |  |  |  |  |

**表3. 调查前两周内病伤情况调查表**

| 被调查成员代码 | | 01 | 02 | 03 | 04 | 05 | 06 |
| --- | --- | --- | --- | --- | --- | --- | --- |
|  | 调查前两周内，您的身体是否有病伤的情况？  ⑴是***(继续询问下列问题）*** ⑵否***（转问表4）*** |  |  |  |  |  |  |

***注：两周病伤指符合下列情况之一者**

调查前14天内：①有就诊、②对病伤有医疗（如服药物或采用推拿按摩热敷等辅助疗法）、③因病伤，休工、休学或卧床一天及以上的情况（老年人明显精神不振、食欲减退或婴幼儿异常哭闹、食欲减退等）

**下列内容询问调查前两周内有患病伤的成员，由调查员从第一列开始按顺序填写，**

**如患有2种及以上病伤，每一种疾病都需要询问，每一种疾病填写一列，成员代码不变。**

| 被调查成员代码 | |  |  |  |  |  |  |  |  |
| --- | --- | --- | --- | --- | --- | --- | --- | --- | --- |
|  | 您患的是什么病或伤？ (填疾病名称) |  |  |  |  |  |  |  |  |
| 92A | （查填疾病代码) |  |  |  |  |  |  |  |  |
|  | 您这次病伤是什么时候开始发病的？  ⑴两周内新发 ⑵急性病两周前开始发病  ⑶慢性病持续到两周内 |  |  |  |  |  |  |  |  |
|  | 两周内，该病伤持续了几天（最长14天）？ |  |  |  |  |  |  |  |  |
|  | 两周内，因该病伤卧床休息了几天（最长14天）？（无卧床，填0） |  |  |  |  |  |  |  |  |
|  | 两周内，因该病伤休工了几天（最长14天）？（无休工，填0） |  |  |  |  |  |  |  |  |
|  | 两周内，因该病伤休学了几天（最长14天）？（无休学，填0） |  |  |  |  |  |  |  |  |
|  | 两周内，您是否因该种病伤就诊过？ ⑴是***（跳问100）***  ⑵否 |  |  |  |  |  |  |  |  |
| 被调查成员代码 | |  |  |  |  |  |  |  |  |
|  | 两周内未就诊的原因（单选）： ***（问完该问题后，跳问116）***  ⑴两周前就医，遵医嘱持续治疗中  ⑵自感病轻 ⑶经济困难 ⑷就诊麻烦 ⑸无时间  ⑹交通不便 ⑺无有效措施 ⑻其它原因 |  |  |  |  |  |  |  |  |
|  | 两周内，为该病伤就诊过几次（次）？ |  |  |  |  |  |  |  |  |
|  | 两周内，为该病伤第一次就诊是在下列哪类医疗机构：  ⑴诊所/村卫生室 ⑵社区卫生服务站 ⑶卫生院  ⑷社区卫生服务中心 ⑸县/县级市/省辖市区属卫生机构  ⑹省辖市/地区/直辖市区属卫生机构  ⑺省/自治区/直辖市属及以上卫生机构 ⑻其它 |  |  |  |  |  |  |  |  |
|  | 您认为此次就诊候诊所花的时间长短如何：  ⑴很短 ⑵短 ⑶一般 ⑷长 ⑸很长 |  |  |  |  |  |  |  |  |
|  | 您认为此就诊机构的环境如何：  ⑴很好 ⑵好 ⑶一般 ⑷差 ⑸很差 |  |  |  |  |  |  |  |  |
|  | 您认为此次就诊医护人员向您解释问题的态度如何：  ⑴很好 ⑵好 ⑶一般 ⑷差 ⑸很差 |  |  |  |  |  |  |  |  |
|  | 您认为此次就诊医护人员向您解释治疗方案的清晰程度如何：  ⑴很好 ⑵好 ⑶一般 ⑷差 ⑸很差 |  |  |  |  |  |  |  |  |
|  | 您认为此次就诊医护人员倾听您述说病情的认真程度如何：  ⑴很好 ⑵好 ⑶一般 ⑷差 ⑸很差 |  |  |  |  |  |  |  |  |
|  | 您对此次为您诊治疾病的医务人员信任程度如何：  ⑴很信任 ⑵信任 ⑶一般 ⑷不信任 ⑸很不信任 |  |  |  |  |  |  |  |  |
|  | 您认为此次就诊的花费如何：  ⑴不贵 ⑵一般 ⑶贵 |  |  |  |  |  |  |  |  |
|  | 您对此次就诊总体满意程度如何：  ⑴满意***（跳问111）*** ⑵一般***（跳问111）*** ⑶不满意 |  |  |  |  |  |  |  |  |
|  | 如有不满意，您最不满意的是什么：（选一项）  ⑴技术水平低 ⑵设备条件差 ⑶药品种类少 ⑷服务态度差  ⑸收费不合理 ⑹医疗费用高 ⑺看病手续烦琐 ⑻等候时间过长  ⑼环境条件差 ⑽提供不必要服务(包括药品和检查) ⑾其它 |  |  |  |  |  |  |  |  |
|  | 两周内，您是否因该病接受了输液治疗？ ⑴是 ⑵否 |  |  |  |  |  |  |  |  |
|  | 两周内，该疾病就诊花费中自己支付了多少钱（元）？  （不包括报销及个人医疗帐户中支出的部分） |  |  |  |  |  |  |  |  |
|  | 两周内，为该病就诊总共花费了多少交通等其它相关费用（元）？ |  |  |  |  |  |  |  |  |
|  | 两周内，您是否因该病到过中医医院就诊？ ⑴是 ⑵否 |  |  |  |  |  |  |  |  |
|  | 两周内，您是否因该病到过综合医院中医科就诊？ ⑴是 ⑵否 |  |  |  |  |  |  |  |  |
|  | 两周内，您是否进行过自我医疗？  ⑴是 ⑵否***（跳问120）*** |  |  |  |  |  |  |  |  |
|  | 您自我医疗，是否使用了药物？ ⑴是 ⑵否***（转问表4）*** |  |  |  |  |  |  |  |  |
|  | 您自我医疗的药物是从哪里来的：（最多可选两项）  ⑴两周内新买的 ⑵家里原有的 ⑶其它 |  |  |  |  |  |  |  |  |
|  |  |  |  |  |  |  |  |
|  | 如自我医疗的药物是两周内新买的，买药自己支付了多少钱（元）？  （不包括报销及个人医疗帐户中支出的部分） |  |  |  |  |  |  |  |  |
|  | 两周内，您是否因该病使用了中药？ ⑴是 ⑵否 |  |  |  |  |  |  |  |  |

**表4. 调查前一年内住院情况调查表**

| 被调查成员代码 | | 01 | 02 | 03 | 04 | 05 | 06 |
| --- | --- | --- | --- | --- | --- | --- | --- |
|  | 近12个月内，您是否有医生诊断需住院而您未住院的情况？  ⑴是　　⑵否***（跳问124）*** |  |  |  |  |  |  |
|  | 共有几次？（同一种疾病医生多次诊断，计为1次） |  |  |  |  |  |  |
|  | 您最近一次需住院而未住院的原因：  ⑴没必要 ⑵无有效措施 ⑶经济困难  ⑷医院服务差 ⑸无时间 ⑹无床位 ⑺其它 |  |  |  |  |  |  |
|  | 近12个月内，您是否因病伤、体检、分娩等原因住过医院？  ⑴是 ⑵否***（转问表5）*** |  |  |  |  |  |  |
|  | 如有住院，住了几次（次）？ |  |  |  |  |  |  |

**下列内容询问调查前一年内有住院经历的成员，由调查员从第一列开始按顺序填写，**

**若住院次数为2次及以上者，每一次住院情况都要询问，每一次住院填写一列，成员代码不变。**

| 被调查成员代码 | |  |  |  |  |  |  |
| --- | --- | --- | --- | --- | --- | --- | --- |
|  | 您这次住院的原因： ⑴疾病 ⑵损伤中毒 ⑶康复 ⑷计划生育  ⑸分娩 ⑹健康体检 ⑺其它 |  |  |  |  |  |  |
|  | 您患的是什么病或伤等? (填疾病名称) |  |  |  |  |  |  |
| 127A | (查填疾病代码) |  |  |  |  |  |  |
|  | 本次住院的入院时间：（年） **（填写4位数字，如：1998）** |  |  |  |  |  |  |
|  | （月） **（填写2位数字，如：07）** |  |  |  |  |  |  |
|  | 您是在下列哪类医疗机构住院的：  ⑴卫生院 ⑵社区卫生服务中心  ⑶县/县级市/省辖市区属卫生机构  ⑷省辖市/地区/直辖市区属卫生机构  ⑸省/自治区/直辖市属及以上卫生机构 ⑹其它 |  |  |  |  |  |  |
|  | 本次住院，您利用中医服务的情况：  ⑴住院机构是中医医院  ⑵住院机构是综合医院中医科 ⑶非中医 |  |  |  |  |  |  |
|  | 等候入院的时间（当天入院填一天）（天）？ |  |  |  |  |  |  |
|  | 本次住院，您是否做过手术? ⑴是 ⑵否 |  |  |  |  |  |  |
|  | 本次住院的天数（天）？ |  |  |  |  |  |  |
|  | 住院期间是否有人在医院陪护您？  ⑴是　　⑵否 |  |  |  |  |  |  |
|  | 如有陪护，最主要是谁陪护您的：  ⑴配偶 ⑵子女 ⑶父母 ⑷孙子女 ⑸兄弟姐妹  ⑹亲戚 ⑺朋友 ⑻保姆 ⑼护工 ⑽其他 |  |  |  |  |  |  |
|  | 本次出院是由于： ⑴病愈医生要求 ⑵病未愈医生要求  ⑶自己要求 ⑷其它原因 |  |  |  |  |  |  |
|  | 如您自己要求出院，原因是：  ⑴久病不愈 ⑵自认为病愈 ⑶经济困难 ⑷花费太多  ⑸医院设施差 ⑹服务态度不好 ⑺医生技术差 ⑻其它 |  |  |  |  |  |  |
|  | 本次住院医药费用总共是多少元? |  |  |  |  |  |  |
|  | 其中：自己支付了多少元？  （不包括报销及个人医疗帐户中支出的部分） |  |  |  |  |  |  |
| 被调查成员代码 | |  |  |  |  |  |  |
|  | 本次住院，所花费的车旅、住宿、伙食、陪护等其它费用合计是多少（元）?  （没有填0） |  |  |  |  |  |  |
|  | 您认为此次住院的病房环境如何：  ⑴很好 ⑵好 ⑶一般 ⑷差 ⑸很差 |  |  |  |  |  |  |
|  | 您认为此次住院医护人员向您解释问题的态度如何：  ⑴很好 ⑵好 ⑶一般 ⑷差 ⑸很差 |  |  |  |  |  |  |
|  | 您认为此次住院医护人员向您解释治疗方案的清晰程度如何：  ⑴很好 ⑵好 ⑶一般 ⑷差 ⑸很差 |  |  |  |  |  |  |
|  | 您认为此次住院医护人员倾听您述说病情的认真程度如何：  ⑴很好 ⑵好 ⑶一般 ⑷差 ⑸很差 |  |  |  |  |  |  |
|  | 您对此次为您治疗疾病的医务人员信任程度如何：  ⑴很信任 ⑵信任 ⑶一般 ⑷不信任 ⑸很不信任 |  |  |  |  |  |  |
|  | 您认为此次住院的医疗花费如何：  ⑴不贵 ⑵一般 ⑶贵 |  |  |  |  |  |  |
|  | 您对此次住院总体满意程度如何：  ⑴满意**（转问表5）** ⑵一般**（转问表5）** ⑶不满意 |  |  |  |  |  |  |
|  | 如有不满意，您最不满意的是什么：（选一项）  ⑴技术水平低 ⑵设备条件差 ⑶药品种类少 ⑷服务态度差  ⑸收费不合理 ⑹医疗费用高 ⑺看病手续烦琐 ⑻等候时间过长  ⑼环境条件差 ⑽提供不必要服务(包括药品和检查) ⑾其它 |  |  |  |  |  |  |

**表5.** 5**岁以下儿童调查表**

**本表由孩子母亲或知情人回答（调查2008年8月15日以后出生的儿童）**

| 被调查成员代码 | | 01 | 02 | 03 | 04 | 05 | 06 |
| --- | --- | --- | --- | --- | --- | --- | --- |
|  | 以下问题由谁回答的：**（调查员选择）**  ⑴母亲　 ⑵父亲 ⑶家庭其他成员 |  |  |  |  |  |  |
|  | 该儿童日常同谁一起生活：  ⑴父母 ⑵祖父母 ⑶哥哥/姐姐 ⑷亲戚 ⑸其他 |  |  |  |  |  |  |
|  | **（询问农村地区）**该儿童父亲是否长期在外务工？ ⑴是 ⑵否 |  |  |  |  |  |  |
|  | **（询问农村地区）**该儿童母亲是否长期在外务工？ ⑴是 ⑵否 |  |  |  |  |  |  |
|  | 孩子是否吃过母乳？ ⑴是 ⑵否***（跳问157）*** |  |  |  |  |  |  |
|  | 孩子纯靠母乳喂养到几个月（月）？***（开始就不是纯母乳喂养填0）*** |  |  |  |  |  |  |
|  | 孩子在几个月大时开始有规律添加辅食（月）？ |  |  |  |  |  |  |
|  | 近12个月内，孩子接受了几次健康体检（次）？（不包括为治疗疾病而做的检查）  ***（没做过填0，并跳问160）*** |  |  |  |  |  |  |
|  | 健康检查时,是否测量过体重? ⑴是 ⑵否 |  |  |  |  |  |  |
|  | 健康检查时,是否测量过身高? ⑴是 ⑵否 |  |  |  |  |  |  |
|  | 孩子有预防接种证吗？ ⑴有 ⑵没有 ⑶不知道 |  |  |  |  |  |  |
| 被调查成员代码 | | 01 | 02 | 03 | 04 | 05 | 06 |
| 查看免疫接种卡并按记录填写接种情况，没有卡的询问接种情况 | |  |  |  |  |  |  |
|  | 是否接种了卡介苗？ ⑴是 ⑵否 |  |  |  |  |  |  |
|  | 共接种了几次百白破疫苗（次）？ |  |  |  |  |  |  |
|  | 共接种了几次脊髓灰质炎疫苗（糖丸）（次）？ |  |  |  |  |  |  |
|  | 共接种了几次含麻疹成分的疫苗（包括麻风、麻腮风、麻腮、麻疹疫苗）？（次） |  |  |  |  |  |  |
|  | 共接种了几次乙肝疫苗（次）？ |  |  |  |  |  |  |
|  | 您对医疗机构为孩子提供预防接种服务的满意程度：  ⑴满意 ⑵一般 ⑶不满意 |  |  |  |  |  |  |

**表6. 15-64岁妇女调查表**

**询问1949年8月16至1998年8月15日出生的妇女**

| 被调查成员代码 | | 01 | 02 | 03 | 04 | 05 | 06 |
| --- | --- | --- | --- | --- | --- | --- | --- |
|  | 近12个月内，您是否做过妇科检查？ ⑴是 ⑵否 |  |  |  |  |  |  |
|  | 近12个月内，您是否做过宫颈涂片检查？ ⑴是 ⑵否 |  |  |  |  |  |  |
|  | 近12个月内，您是否做过乳腺检查？ ⑴是 ⑵否 |  |  |  |  |  |  |
|  | **（农村地区询问）**当您在乡镇卫生院希望由女性医生提供服务时，您是否能够得到？  ⑴是 ⑵否 ⑶没想过 ⑷不知道 |  |  |  |  |  |  |
|  | **（农村地区询问）**您丈夫是否长期在外务工？ ⑴是 ⑵否 |  |  |  |  |  |  |
|  | 您曾经怀孕过几次（次）？ ***（未曾怀孕填0，结束该成员调查）*** |  |  |  |  |  |  |
|  | 您曾经生过几个孩子（人）？ ***（未曾分娩填0，结束该成员调查）*** |  |  |  |  |  |  |
|  | 您最后一次分娩的时间: （年）**（填写4位数字，如：1998）** |  |  |  |  |  |  |
|  | （月）**（填写2位数字，如：07）** |  |  |  |  |  |  |
| **本表下面问题询问2008年8月15日以后有分娩的妇女** | | | | | | | |
|  | 您最后一个孩子的性别： ⑴男 ⑵女 |  |  |  |  |  |  |
|  | 您怀最后一个孩子期间，做过几次产前检查（次）？(***从未做过填0***，***跳问182题***) |  |  |  |  |  |  |
| 被调查成员代码 | | 01 | 02 | 03 | 04 | 05 | 06 |
|  | 此次怀孕产前检查时，是否做过抽血检查？ ⑴是 ⑵否 |  |  |  |  |  |  |
|  | 是否测量过血压？ ⑴是 ⑵否 |  |  |  |  |  |  |
|  | 是否查过尿？ ⑴是 ⑵否 |  |  |  |  |  |  |
|  | 是否做过B超检查？ ⑴是 ⑵否 |  |  |  |  |  |  |
|  | 孩子是如何出生的： ⑴自然分娩***（跳问184题***) ⑵剖宫产 |  |  |  |  |  |  |
|  | 如为剖宫产，主要是谁提议的： ⑴自己要求 ⑵医生建议 ⑶其它人建议 |  |  |  |  |  |  |
|  | 您是在哪里分娩的：  ⑴县及以上医院 ⑵妇幼保健机构 ⑶乡镇街道卫生院  ⑷社区卫生服务中心 ⑸卫生室/所/站 ⑹家中 ⑺其它 |  |  |  |  |  |  |
|  | 如在家中分娩，接生者是谁： ⑴乡及以上医生 ⑵村医生 ⑶专职接生员  ⑷非专职接生者 ⑸家人自接 ⑹其它 |  |  |  |  |  |  |
|  | 小孩出生时体重为多少克？ |  |  |  |  |  |  |
|  | 分娩费用总共是多少元？ |  |  |  |  |  |  |
|  | 其中，自己支付了多少元？（没有填0）  （不包括报销及个人医疗帐户中支出的部分） |  |  |  |  |  |  |
|  | 产后42天内，您接受产后访视的次数（次）？（没有填写0） |  |  |  |  |  |  |

**该住户家庭成员全部调查完毕后，填写下表（结果填在右侧空格中）**

| 1 | 该住户调查前半年内常住人口数（人） |  |
| --- | --- | --- |
| 2 | 其中：15-64岁妇女数（人） |  |
| 3 | 5岁以下儿童数（人） |  |
| 4 | 调查前两周内患病伤人数（人） |  |
| 5 | 调查前一年内住院人数（人） |  |
| 6 | 该住户是： ⑴初次调查 ⑵再次调查 |  |
| 7 | 该住户是： ⑴第一次抽中户 ⑵替补调查户 |  |
| 8 | 调查持续时间（分钟） |  |
