# Supplementary material for: Patient Satisfaction with Hospital Inpatient Care: Effects of Trust, Medical Insurance and Perceived Quality of Care
Source: PLoS One. 2016 Oct 18;11(10):e0164366. doi: 10.1371/journal.pone.0164366 (PMC5068749; doi:10.1371/journal.pone.0164366)
Supplement: S2 Appendix — (DOC) [file pone.0164366.s002.doc]

**半结构式访谈提纲——医患信任的影响因素**

**致辞**

尊敬的被访谈者：

您好！我们是哈尔滨医科大学医患关系研究小组的成员，此次访谈的目的是了解您对医患信任的影响因素的看法，研究结果将为重构和谐医患关系提供参考与依据。访谈信息将仅被用于统计分析，感谢您的时间与配合！

**基础信息**

姓名： _______________ 性别： _____

年龄： _____ 学历： ____________

工作单位： _______________________________________________

工作职位： _______________________________________________

**问题：**

1. 您认为中国目前的医患信任状况如何？

[医患信任对医患关系的影响程度如何？]

1. 您认为在医患个人互动层面医患的信任情况如何？

[在这一层面影响患者信任度的因素有哪些？从患方的角度来看？从医方的角度来看？]

1. 您认为在医疗服务提供层面医患的信任情况如何？

[在这一层面影响患者信任度的因素有哪些？从患方的角度来看？从医方的角度来看？]

1. 您认为在系统层面医患的信任情况如何？

[在这一层面影响患者信任度的因素有哪些？从患方的角度来看？从医方的角度来看？]

1. 除了上述三个维度外，您认为还有哪些维度应被考虑？

[在这一层面影响患者信任度的因素有哪些？从患方的角度来看？从医方的角度来看？]

1. 您认为上述分析维度之间的关系是怎样的？
2. 关于此次访谈您有什么意见或建议吗？

访谈时间：______________________

核实时间：______________________

访谈人员（签名）：_______________

调查指导员（签名）：_____________
